# A review on drug repurposing in COVID-19: from antiviral drugs to herbal alternatives

**DOI:** 10.1186/s43141-022-00353-0

**Published:** 2022-05-24

**Authors:** Abas Sezer, Mervisa Halilović-Alihodžić, Annissa Rachel Vanwieren, Adna Smajkan, Amina Karić, Husein Djedović, Jasmin Šutković

**Affiliations:** 1 Genetics and Bioengineering, International University of Sarajevo, Sarajevo, Bosnia and Herzegovina; 2 Fakultät Chemie und Pharmazie, Ludwig-Maximilians-Universität München, Munich, Germany

**Keywords:** COVID-19, Drug repurposing, Herbal medicine, Remdesivir, Ivermectin

## Abstract

**Background:**

COVID-19 is an illness caused by severe acute respiratory syndrome coronavirus 2. Due to its rapid spread, in March 2020 the World Health Organization (WHO) declared pandemic. Since the outbreak of pandemic many governments, scientists, and institutions started to work on new vaccines and finding of new and repurposing drugs.

**Main body of the abstract:**

Drug repurposing is an excellent option for discovery of already used drugs, effective against COVID-19, lowering the cost of production, and shortening the period of delivery, especially when preclinical safety studies have already been performed. There are many approved drugs that showed significant results against COVID-19, like ivermectin and hydrochloroquine, including alternative treatment options against COVID-19, utilizing herbal medicine.

**Short conclusion:**

This article summarized 11 repurposing drugs, their positive and negative health implications, along with traditional herbal alternatives, that harvest strong potential in efficient treatments options against COVID-19, with small or no significant side effects. Out of 11 repurposing drugs, four drugs are in status of emergency approval, most of them being in phase IV clinical trials. The first repurposing drug approved for clinical usage is remdesivir, whereas chloroquine and hydrochloroquine approval for emergency use was revoked by FDA for COVID-19 treatment in June 2020.

## Background

Coronavirus disease 2019 or shortly COVID-19 is an illness caused by severe acute respiratory syndrome coronavirus 2 [1]. It started as an epidemic in China at the end of 2019 in China, Wuhan [2]. By now, 229E, NL63, OC43, and HKU1 are four of the seven most known coronaviruses identified in humans. They may induce different respiratory infections such as cold, Middle East respiratory syndrome (MERS), severe acute respiratory syndrome (SARS), or the novel coronavirus (COVID-19) disease [3]. Due to COVID-19 rapid spread, in March 2020 the World Health Organization (WHO) declared COVID-19 pandemic, and until now being linked to more than 5 million deaths worldwide [1]. Since the outbreak of pandemic many governments, scientists, institutions started to work on vaccines and treatments. Due to high rate of deaths, doctors used wide range of drugs for treatment which in some cases led to devastating consequences, but in other cases different treatments showed excellent results. In this regard, drug repurposing seems like an excellent opportunity to find an effective drug against COVID-19 [4]. Considering the costs and period needed to produce a new drug, repurposing the old drugs have become an attractive alternative. The process itself is based on the identification of new uses of already existing drugs, thus generating a promising treatment option in a remarkably shorter time [5]. This review focuses on different types of drugs, as well as on drug candidates that showed promising results against COVID-19 infection, based on an electronic search using PubMed and Google Scholar, including alternative options found in herbal medicine.

## Main text

### Drug repurposing in correlation to COVID-19

COVID-19 created quick and unprecedented difficulties to healthcare systems in nearly every country on the planet [6]. However, the rapid discovery of effective COVID-19 drugs is not assured. Developing and approving an effective antiviral medication from scratch is a dangerous, expensive, and time-consuming process. Drug repurposing or repositioning is an appealing kind of drug discovery since it recycles old treatments to treat new diseases and save shelved drugs [7]. Repurposing can help find new treatments for diseases at a lesser cost and in a shorter period of time, especially when preclinical safety studies have already been performed. To date, the most well-known repurposed medications have been discovered either by chance or based on specific pharmacological insights or using experimental screening platforms [8]. Omics technologies, involving computational methods developed novel approaches for medication repositioning [9]. With the increase in drug-related data and open-data initiatives, a new set of repositioning methods and approaches has arisen, involving the integration of data from pharmacological, genetic, chemical, or clinical data [9, 10]. These technologies opened a door to propositions of new pharmacological medications, increasing the success rate from 30 to 75% [10, 11].

Before it can be considered for progression through the development pipeline, drug repurposing for COVID-19 goes through the same three steps as all other drug repurposing projects: mechanistic evaluation of the drug effect in preclinical models, candidate drug identification, and evaluation of candidate drug’s efficacy in phase II clinical trials [12]. The first of these three processes is the most important: finding a COVID-19 medication with a high repurposing potential [5].

Virus-related targets and host-related targets are two types of therapeutic targets based on collective mode of pathogenicity [13]. If an antiviral medicine has the capacity to target a specific viral replication route while also being effective against other viruses, it may be beneficial against COVID-19 [14]. Patients with SARS-CoV-2 infection are currently largely treated by repurposing existing drugs, which differ depending on the patient’s symptoms (Table 1).

**Table 1:** COVID-19 correlated repurposing drugs in clinical trials

***Drug name***	***Original indication***	***New indication/remark***	***Clinical trial stage***
Chloroquine	In treatment for systemic lupus erythematosus and rheumatoid arthritis	Emergency approval for COVID-19 treatment revoked by FDA (June 2020)-withdrawn	Phase III
Hydrochloroquine	In treatment for rheumathoid arthritis and Sjorgren’s syndrome	Emergency approval for COVID-19 treatment revoked by FDA(June 2020)-withdrawn	Phase III
Ivermectin	For strongyloidiasis and onchocerciasis treatment	COVID-19 treatment	Phase III
Interferon alfa (IFN-α)	Cancer immunotherapy	COVID-19 treatment	Phase II
Fluvoxamine	In treatment for obsessive-compulsive disorder	COVID-19 treatment	Phase III
Molnupiravir	Anti-viral drug	Emergency approval for COVID-19 treatment (December 2021)/UK Regulatory Agency approval	Phase III/clinical use
Sofosbuvir	Chronic hepatitis C	COVID-19 treatment	Phase IV
Ebselen	Demonstrating cytotoxicity against yeast, fungi, and bacteria	COVID-19 treatment	Phase II
Favipiravir	Antiviral activity against influenza, yellow fever and ebola	Emergency approval for COVID-19 treatment (May, 2020)	Phase III/clinical use
Ribavirin	Chronic hepatitis C infection	COVID-19 treatment	Phase II
Remdesivir	Antiviral activity against paramyxoviruses, flaviviruses and coronaviruses.	COVID-19 treatment/FDA approved in October 2020	Clinical use

Drug clinical stage information available at: www.clinicaltrial.gov

**Fig. 1:**
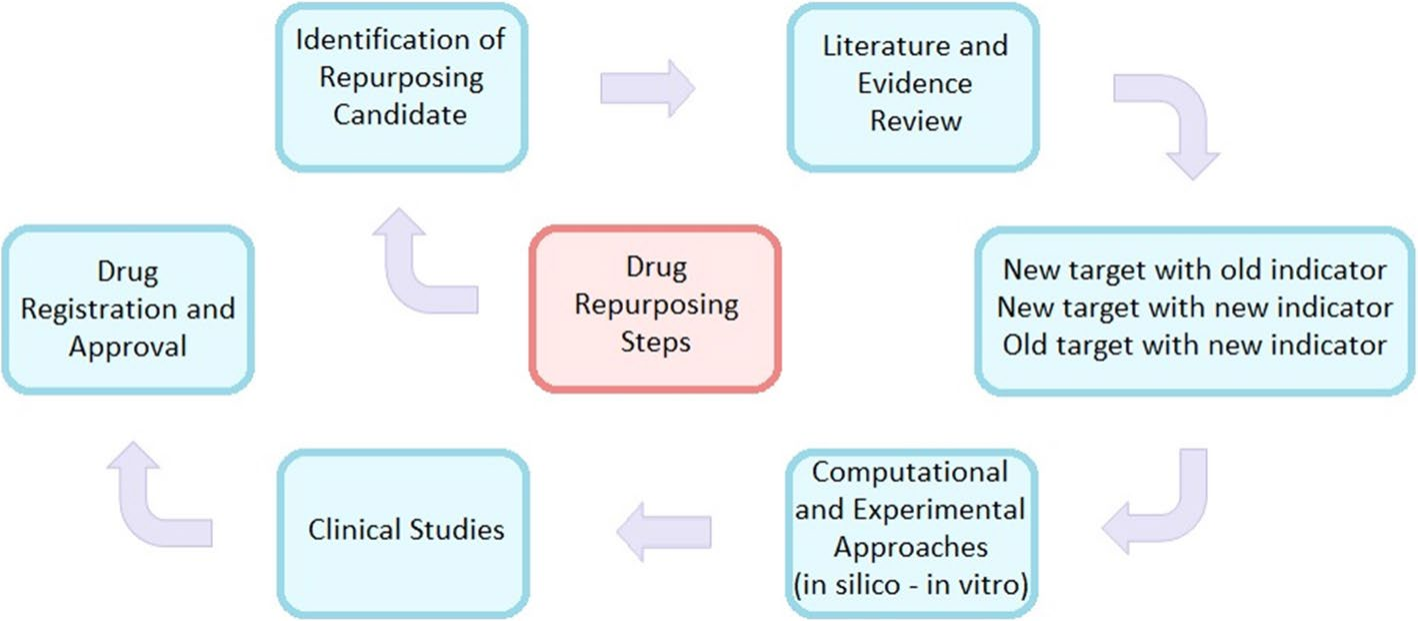
Conceptual diagram of drug repurposing steps

Secondary infections, antibiotics and antiviral medications are used to treat ARDS (acute respiratory distress syndrome). Among the available treatments of COVID-19 are antibiotics, RNA synthesis inhibitors, antiviral drugs, traditional herbal medicines, and neuraminidase inhibitors [15]. Nonetheless, carefully conducted clinical trials are needed to verify the effectiveness of various therapy regimens [7]. According to recent research, developing a new proven medicine costs billions of dollars and takes an average of 9–12 years to bring to market [16]. Computational techniques, including molecular docking, drug signature matching, genome-wide association studies, and network analysis are all common computational drug repurposing methodologies to medication repurposing [12, 17]. Step in drug repurposing is presented in Fig. 1.

Recent improvements in artificial intelligence (AI) and machine learning have also been emphasized for COVID-19 drug repurposing, with numerous research utilizing these approaches reporting encouraging findings [6, 9, 18].

### Available treatments for COVID-19

#### Chloroquine

Chloroquine (CQ) is 4-aminoquinoline compound, used as medication to treat diseases and disorders such as systemic lupus erythematosus, rheumatoid arthritis and different skin conditions [19]. National guidelines in China suggested CQ for treatment of COVID-19 patients. Two main reasons for this suggestion were that this medicine was previously recorded for effective cure against SARS-CoV-1 and some in vitro experiments in China, including more than 100 patients, showed that CQ could inhibit viral replication [20]. Results from these tests showed reduction in duration of illness and improvement in pneumonia infection. A different study from March 2020, tested 36 patients with different COVID-19 symptoms such as upper and lower respiratory tract problems and some of patients were without symptoms. Results showed that patients in the sixth day of treatment tested negative for the virus [21, 22]. The chloroquine raises pH level in host cell lysosomes and that affects virus receptor linking as well as connection with the glycosylation of SARS-CoV-2 receptors [23]. Before using CQ in combination with other drugs, patients need to do electrocardiography test. This step is necessary because CQ in combination with other medications can lead to extension of the corrected interval of the heart [24]. High doses of CQ were reported to cause several side effects, but prescribed and controlled doses of CQ had fewer side effects [25]. These side effects include visual problems such as diplopia, retinal toxicity and loss of vision. High dose can also cause problems such as paranoia and hallucinations [25, 26]. American study tested QT-interval in COVID-19 patients that were treated with CQ/azithromycin combination, and they came to conclusion that electrical activity of the heart went up. Some of the patients were in danger of sudden cardiac death caused by malignant arrhythmia [27]. In Brazil scientists compared the effect of high doses and small doses of CQ in patients, where 39% of patients in the high CQ dose group and 15% patients in small CQ dose group died [28]. This study suggests that the right dose of CQ is still not determined in COVID-19 patients. Although in March 2020, the FDA has approved the emergency in treatment of COVID-19, this decision is withdrawn in June 2020, due to large number of reports of side effect. Therefore, further investigation of CQ effects on COVID-19 patients is highly recommended.

#### Hydrochloroquine

Hydrochloroquine is known as a metabolite of chloroquine. Their mechanisms of action and structure are similar. Hydrochloroquine is less toxic, and it is used for treatment of diseases such as rheumathoid arthritis, Sjorgren’s syndrome, and juvenile idiopathic arthritis [29]. The reason why is hydroxychloroquine proposed as a potential treatment for COVID-19 is based on its SARS-CoV-2 antiviral activity. Data obtained from certain studies it is effective in reduction of viral loads [30]. Based on the study conducted in France, hydrochloroquine seems to be effective in clearing viral nasopharyngeal carriage in 3 to 6 days in patients diagnosed with COVID-19 [21]. Based on evidence obtained from central clinical task force from Korea, it is reported that they treated 27 cases of COVID-19 using different antiviral drugs. Among those drugs effectively hydrochloroquine showed significant effects, in 400 mg doses per day [31]. Hydroxychloroquine was also recommended in China, Netherlands, France, South Korea, and Italy [32]. Many studies showed that in vitro inhibition of some viruses by hydroxychloroquine is possible; however its full anti-viral activity has not been proven in any virus model in vivo [33]. In 2021, Rocha et al. concluded that hydroxychloroquine (with chloroquine) is correlated to G6DP gene, whose enzyme is the crucial in production of nicotinamide adenine dinucleotide phosphate NADPH. This study concluded that G6DP deficiency must be considered while treating COVID-19 patients with CQ/HCQ treatment, since this deficiency was most common associated among the African population [34]. Hydrochloroquine is usually considered safe; however, some side effects have been reported such as gastrointestinal discomfort as one of the most common side effects, but there are others as well such as allergic reactions, respiratory distress with patients with proximal muscle weakness, hearth rhythm problems, liver and kidney problems, hypoglycemia, and retinopathy [35, 36]. However, the use of chloroquine and hydrochloroquine resulted in diverse and contrary results, different trials leading to different conclusion. Therefore, the suggestion by WHO is to conduct more research studies that utilize CQ in use with COVID-19 patients [37].

#### Ivermectin

There are many studies suggesting that ivermectin has antiviral properties against RNA viruses such as HIV, dengue, influenza, and SARS-CoV-2 [38]. Research conducted in Australia suggests that one of the drugs that inhibit SARS-CoV-2 replication is ivermectin [39]. Based on antiviral properties of ivermectin many studies are conducted in order to prove its effect on the COVID-19. One of the studies conducted in Argentina shows that health care workers involved in the care of patients diagnosed with COVID-19 treated with the combinatorial treatment of ivermectin and carrageenan, so-called IVERCAR, were not tested positive during the 14-day treatment period [40]. Randomized controlled trials (RCTs) were conducted in Egypt. There were 340 patients positive for COVID-19. After administration of ivermectin there were significant decrease in symptoms of COVID-19 [41]. In addition, study conducted in Bangladesh included 72 patients. Their study showed that during 5-day course, COVID-19 treatment with ivermectin resulted in earlier clearance of the virus. However, it is important to mention that early intervention with ivermectin was crucial in their study, and it surely has huge impact in limitation of viral replication [42]. Docking study and molecular dynamic simulations showed that ivermectin inhibited more than 85% of enzymatic activity of 3 CL protease. That is the highest percentage among 10,755 different compounds. 3CL protease or 3-chymotrypsin like protease is crucial for development of antiviral drugs. It has important role in generating functional and non-structural viral proteins [43]. When it comes to safety of ivermectin, few studies report that majority of side effects were mild such as: rash, fever, itching, and headache [44]. A combined results of a study of 50,000 patients infected with Loa loa, and treated with ivermectin, shows that serious events related to ivermectin occurred in less than 1% of patients [45]. Two cases of liver injury were reported in patients that were using ivermectin, both cases were solved without any need for treatment [46]. Additionally, ivermectin could also be neurotoxic, however, among 4 billion doses only 28 cases were reported [47].

#### Interferon α

Interferons (IFNs) are proteins, cytokines important for immune response whose role is to act on innate and adaptive immunity. They are cell signaling proteins that are expressed in response to viral infections, tumors, and many other infectious agents. We can say that they act as “first line” of defense against those agents and that they can provide a protective effect in the early phases of the disease, before viral peak. IFNs are used alone or in combination with other drugs and treatments. Approved by 22 different regulatory agencies, IFNs show promising clinical result [48, 49]. In correlation to SARS-CoV-1 and MERS-CoV outbreaks, studies have shown that interferon α (IFNα) had beneficial effects on lung problems in hospitalized patients with SARS-CoV [50, 51]. Its antiviral activity and role in regulating inflammatory factors shows that IFN-α therapy is promising idea to manage several stages of COVID-19 [52]. IFN-α was combined with lopinavir, resulting in reduction of hospitalization duration, and improved viral clearance in COVID-19 patients [53]. In 2021, a group of scientists used auto-antibodies against IFNs, and the results showed that this treatment is less effective against COVID-19 [54]. A different study showed that IFNα mono-therapy, in combination with ARB drugs was more effective in viral clearance and in decreasing hospitalization days [55]. Effectiveness of three different combinations was tested in patients with mild to moderate COVID-19. However, in combination with ribavirin/lopinavir the effect was not significant [56]. Combination of IFN-α and antiviral drugs, if used in the first days of virus, may lead to a rapid suppression of high viral load also makes antiviral response stronger. There is still small number of studies, but until now research showed promising path for IFNα treatment. Many factors affect IFNα efficacy, including disease severity and treatment time. What is interesting is that no significant drug reactions or IFNα side effects were reported [52].

#### Fluvoxamine

Fluvoxamine belongs to antidepressant drug family, such as selective serotonin reuptake inhibitor (SSRI) and serotonin-norepinephrine reuptake inhibitor (SNRI), which are shown to be associated with a reduced risk of clinical deterioration in SARS-CoV-2-infected patients [57]. In 2020, a study presented that the SSRI fluvoxamine could prevent clinical deterioration in early-stage COVID-19 outpatients [58]. Another trial from Brazil, including more than 9000 patients, showed that this cheap antidepressant decreased the number of hospitalized patients, using 100 mg twice daily for 10 days [59]. This huge study showed that treatment with fluvoxamine will reduce the need for extended emergency room observation or hospitalization. Although its mechanism of action in the COVID-19 context is uncertain, fluvoxamine was examined as a possible treatment because of its antiviral and anti-inflammatory effects [60]. Further, fluvoxamine is tested as a prophylactic drug, for early-stage SARS-CoV-2-infected patients and as a potential anti-inflammatory drug, in vitro and in vivo models [57, 61]. Side effects are not yet reported in adults, only in maternal use of fluvoxamine on breastfed infants. However, these studies are limited [62].

#### Molnupiravir

Molnupiravir is one of the newest known oral antiviral drugs that have been recently tested in COVID-19 [63]. The first country that has approved molnupiravir is the UK, an anti-COVID-19 drug developed by the US company Merck and which should be the main weapon in the fight against the pandemic. Molnupiravir is approved for people with mild to moderate symptoms who have at least one risk factor for developing a serious illness (obesity, the elderly, diabetes, and heart disease) [64]. Antiviral drugs such as molnupiravir reduce the virus’s ability to replicate, thereby slowing the disease. Phases 1, 2, and 3 of clinical trials resulted in a significantly lower risk of hospitalization or death in adults experiencing mild or moderate COVID-19 [65]. A study conducted on non-hospitalized COVID-19 patients, found a significant reduction in the risk of hospital admission or death by 50% [66]. Molnupiravir is prescribed to patients within a few days after a positive test. In addition, molnupiravir is the first oral antiviral drug that is directly effective in attenuating viral RNA and SARS-CoV-2 in the nasopharynx, which is well tolerated and with good safety [67].

#### Sofosbuvir

Sofosbuvir is a known and approved drug used for treatment of hepatitis C (HCV), capable of suppressing other families of positive-strand RNA viruses [68]. In 2020, a study from Iran examined the effects of sofosbuvir in combination with hydroxychloroquine and other drugs, showed significant result in decreasing the number of patients experiencing fatigue and dyspnea in COVID-19 patients [69]. Other similar studies included relatively small testing groups, where sofosbuvir was compared with daclatasvir, plus placebo as a control group. Those receiving sofosbuvir/daclatasvir-based treatment was associated with numerically shorter length of hospital stay than control group, but the difference did not reach statistical significance [68, 70]. It is shown that sofosbuvir offers low side effects, significant efficacy, short administration period, good tolerability, and high healing rate [71].

#### Ebselen

Ebselen is an organoselenium, antioxidant compound, exhibiting anti-inflammatory and antiviral activities against HIV, hepatitis, Zika virus and influenza [72]. Ebselen may serve as lead compound for drugs targeting COVID-19 [73]. Further, ebselen is known to be effective in prevention of noise-induced hearing loss and bipolar disorder, currently being studied in a phase-2 trial [74, 75]. It is already presented with potent antiviral activity against SARS-CoV-2 via MPRO inhibition, as the main protease of SARS-CoV-2 [76]. Similar activity is also seen in other in vivo and clinical studies, concluding that its repurposing against SARS-CoV-2 is a reasonable option [73]. A recent study confirmed that Ebselen can protect organisms from various human diseases through antioxidant, anti-inflammation, and anti-lipoperoxidation bioactivities, with mild side effects reported so far [77], as it is proposed to be a stable lithium mimetic, used to treat bipolar disorders [78].

#### Favipiravir

Favipiravir is a prodrug that possesses antiviral and anti-influenza properties as it can treat influenza A, B, and C, as well as other RNA viruses such as Yellow fever and Ebola [79]. It became a legal drug in 2014 in Japan to treat influenza and it is used in other countries as well [80]. One study has shown that 38 out of 60 patients demonstrated viral clearance after 4 days. On the fifth day, there was double number of patients that gave negative results for SARS-CoV-2 by PCR. Not only do patients recover quickly, but favipiravir relieve symptoms such as cough and fever [81]. Another study of 96 patients between April and August 2020 found that they stayed less in the hospital and required less mechanical ventilation [82]. Also, it may decrease levels of IL-6 and other lymphocytes which prevent cytokine storm [83]. Even though favipiravir may treat COVID-19 effectively, it may still introduce some side effects. One is that it can affect GI tract causing gastrointestinal illnesses such as nausea and vomiting [84]. Favipiravir is not recommended for pregnant women and lactating women because it may introduce teratogenicity effects. This may disrupt the growth of embryo during pregnancy leading to birth defects. It is therefore recommended for people of reproductive age to take contraceptive for at least 1 week or until favipiravir is no longer in the system [85].

#### Ribavirin

Ribavirin associates are known as antiviral drugs indicated as a therapy for chronic hepatitis C. Ribavirin is a guanosine-like substance, which not only inhibits DNA and RNA virus reproduction as well as RNA capping, nevertheless, increases viral RNA instability [86]. Until now, ribavirin is used as an antiviral medication in combination with an interferon drug (e.g., Pegasys or PEG-Intron) to treat people with HCV who never had an interferon previously. It works by preventing the HCV virus from spreading throughout the body (Drugs, H., 2021). Ribavirin has shown efficacy against MERS-CoV both in vitro and in vivo, and coupled with interferon alfa resulted in virologic clearance and survival. Some studies investigated the utilization of ribavirin in hospitalized patients with corona infection, in combination with lopinavir/ritonavir or interferon, where the data showed that there is no significant increase in the chance of clinical improvement [87]. Ribavirin was approved in China to treat COVID-19 in combination with interferon alfa or lopinavir-ritonavir. However, ribavirin alone was not statistically correlated with the health improvements of adult patients with severe COVID-19 [78]. A recent study done at the Beijing Ditan Hospital (China), showed that no significant clinical benefit was observed from treatment with interferon alfa-2b, together with ribavirin [20].

#### Remdesivir

Antiviral efficiency of remdesivir has been known before, effectively treating in vitro and animal models of SARS and MERS corona viruses [88], and Ebola as well [89]. In 2020, together with interferon β, remdesivir was shown to have superior antiviral activity compared to other antiviral drugs against MERS-CoV in vitro [90]. Remdesivir drug is shown to strongly inhibit SARS-CoV-2 replication and reduces the general viral load within the body in infected animals. Further, it mitigates mild symptoms, reduces the pathological process, and improves pulmonary lesions [91]. In 2020, the World Health Organization conducted a clinical trial and included remdesivir, in an attempt to find an effective treatment for COVID-19 [92]. In this regard, in the USA, the patients who been treated with remdesivir in emergency protocols against COVID-19 showed a significant recovery, where a 35-year-old patient showed significant improvement of his health condition after 7 days of treatment [93]. In October 2020, remdesivir became officially the first approved repurposing drug by the Food and Drug Administration (FDA) agency, for the treatment of COVID-19 [94].

### Traditional medicine in COVID-19 treatment

Herbal medicine can be used to treat COVID-19, as well as to manage its symptoms and prevent it. It is also important to get enough vitamins and nutrients in our body. For instance, vitamin D decreases the risk of a person getting COVID-19 and influenza. Many of these herbs and foods such as *aloe vera* tend to possess immunomodulatory and antiviral activities. This means that cytokines are stimulated and consequently lymphocytes are induced, actions of macrophages are enhanced and there is an increase in the amount of natural killer cells [95]. Some Chinese products such as *Shen Fu Injections* and *Re Du Ning* Injections may prevent a cytokine storm by reducing the amount of IL-1β, IL-6, and other cytokines*. Qingfei Paidu* is found to target the lungs and the spleen, thereby reducing inflammation, and provides a stronger immune system [96].

During this pandemic, Chinese herbal medicine was used successfully to prevent and treat COVID-19. A treatment program using Chinese herbal medicine was set by the *National Administration of Traditional Chinese Medicine*. On January 24th, 2020, the first patient recovered from COVID-19 and was released from the hospital following this treatment program [97]. On January 27th, 2020, a role book for the diagnosis and treatment of pneumonia caused by COVID-19 was published by the National Health Commission of the People’s Republic of China (PRC) [98]. This book includes guidelines and recommendations, with an updated list of traditional Chinese herbal medicine and how these Chinese herbs can be combined with western medicine [96]. It is estimated, that in China, at least 85% of people took Chinese herbal medicine to treat COVID-19 [99]. While patients were recovering from COVID-19 in Wuhan, there, two medicines were recommended and those are *Ginseng and Shengmai san*. *Ginseng* provides faster recovery while *Shanghai san* improves the function of the heart and the circulation of blood [100]. A study showed that these herbal medicines targets ACE_2_ receptors, preventing SARS-CoV2 from entering the cells. Also, the ACE_2_ receptor may be expressed in the gastrointestinal tract which means herbs can be taken orally and undergo intestinal absorption. It is believed that the large intestine and lung meridian line are connected according to the theory of Huang Di Nei Jing. It is based on the balance of fluids in the body, while pulmonary cells exchange _CO2_ and O_2_ [101].

Another herbs, as *Ma xing shi gan* and *Gancao ganjiang* may be used to treat the early stage of a COVID-19 infection because both can treat asthma and aid in proper breathing [100]. Further, *Qingfeipaidu*, *Sheganmahuang*, *Maxingshigan decoction*, *Jinhuaqinggan granules*, and *Lianhuaqingwen* capsules may be used in the treatment-phase of COVID-19. *Qingfeipaidu decoction* includes 21 components and was found to be 92% effective in 101 patients by eliminating lung inflammation, preventing a cytokine storm, and detoxifying the lungs [102]. *Sheganmahua*ng decoction has 9 herbs and is used to treat asthma since it regulates the CD4+/CD8 + ratio of T cells and the expression of IL-5 and IL-10. It also suppresses TLR-4, TSLP, and NF-κB found in lung tissue and IL-17A, TNF-α, and IL-6. This aids in eliminating airway restriction and prevents the damage of the lungs [103]. *Maxingshigan* decoction provides a strong immune system by increasing levels of IL-4 and IL-2 and suppresses the amount of TNF. It may target IL-6, MAPK-1, and other substances [100]. *Lianhuaqingwen* capsules have 11 herbs that manage symptoms such as fever, runny nose, cough, muscle ache, headache, and so on. It has antibacterial, anti-inflammatory, and antiviral effects, and it was found that it shortens the time for treating patients and improves the lifespan of patients. It eliminates replication of SARS-CoV2 [104]. *Jinhuaqinggan* granules treat symptoms such as fever, running nose, sore throat and so on. It is found that it may treat pneumonia and influenza A in mice, thereby it prevents the damage of lungs and inflammation [105]. In India, there is an ancient medical system called Ayurveda and like traditional Chinese medicine (TCM), which provides a holistic and immunotherapy approach, uses different herbs or plants that cure various illnesses and improves the immune system. There are several plant systems tried in treatment of COVID-19, and these are: *Mangifera indica*, Guduchi (*Gilu*, *Giloy*, *Tino sporacordifolia*), A*shwagandha/Withania somnifera*, *Kukum*, *Saffron*, *Rasona*, *Adraka or Ginger*, *Terminalia chebula*, *Piper longum*, *Ocimum sanctum*, *Albizia lebbek*, and *Centella asiatica*. These drugs are currently undergoing clinical trials suggesting that they are reassuring candidates for treating COVID-19 and managing its symptoms, as well as preventing this illness [26]. However, there is concern about using Chinese herbal medicine. Some of these products are found to be contaminated with unknown materials from animals or plants, pesticides or sulfites and this may lead to severe allergies or asthma. Also, if wrong herbs are used, this may lead to organ disruption [106]. Therefore, it is important that the safety, quality, and efficacy of these herbs are determined [107]. Overall, the Chinese traditional medicine, especially in combination with Western medicine, provides an alternative treatment option for COVID-19.

## Conclusion

A sudden outbreak of COVID-19 pandemic has forced the scientists around the world to find a quick and the most adequate COVID-19 treatment. Thus, different drugs have been utilized, but not necessarily each drug had a positive effect. Due to the difficulties that may arise from producing new drugs, drug repurposing has been used as a suitable alternative. Drug repurposing takes advantage of already existing drugs that are approved for certain diseases and test their efficiency for new diseases. In order to be used, the drug must first go through preclinical tests, then clinical trials to test for its efficacy. However, apart from the repurposed drugs belongs to antiviral, antibiotic, and cytokine types. Many studies suggest that ivermectin, chloroquine, and hydroxychloroquine have antiviral, antioxidant, anti-inflammatory, and cytoprotective properties against RNA viruses, but still undergoing final clinical trials. Some multipurpose drugs are under clinical trials as well, most known to treat sickness, diabetes, and heart diseases. Among them, molnupiravir showed effective action against COVID-19, therefore being authorized by the UK Regulatory agency and the FDA (for emergency use). Currently, the only repurposing drug approved by FDA for COVID-19 treatment so far is remdesivir, a strong antiviral drug. Herbal medicine has also been used as a treatment against COVID-19 infection, supported with sufficient vitamin and mineral intake. Different Chinese and Indian herbs were used in traditional medicine, have positively influenced the treatment of patients and quicker recovery. Among plants, *aloe vera* and several Chinese herbs, like *ginger* and *safron*, have shown to have strong immunomodulatory and antiviral properties, but in the early stage of COVID-19 infection. Drug repurposing against COVID-19 has great potential, offering excellent candidates for an effective fight against this virus.

## Data Availability

Not applicable

## Abbreviations

COVID-19: Coronavirus disease of 2019
SARS-CoV: Severe acute respiratory syndrome coronavirus
MERS-CoV: Middle East respiratory syndrome coronavirus
HCV: Hepatitis C virus
HIV: Human immunodeficiency virus
IFN-α: Interferon α
INF-β: Interferon β
NADPH: Nicotinamide adenine dinucleotide phosphate
G6DP: Glucose-6-phosphate dehydrogenase deficiency
CQ: Chloroquine
HCQ: Hydrochloroquine
